# A new efficient method to detect genetic interactions for lung cancer GWAS

**DOI:** 10.1186/s12920-020-00807-9

**Published:** 2020-10-30

**Authors:** Jennifer Luyapan, Xuemei Ji, Siting Li, Xiangjun Xiao, Dakai Zhu, Eric J. Duell, David C. Christiani, Matthew B. Schabath, Susanne M. Arnold, Shanbeh Zienolddiny, Hans Brunnström, Olle Melander, Mark D. Thornquist, Todd A. MacKenzie, Christopher I. Amos, Jiang Gui

**Affiliations:** 1 Quantitative Biomedical Science Program, Geisel School of Medicine, Dartmouth College, Hanover, NH 03755 USA; 2 Department of Biomedical Data Science, Geisel School of Medicine, Dartmouth College, One Medical Center Dr., Lebanon, NH 03756 USA; 3 Institute for Clinical and Translational Research, Dan L. Duncan Comprehensive Cancer Center, Baylor College of Medicine, Houston, TX 77030 USA; 4 Unit of Nutrition and Cancer, Catalan Institute of Oncology (ICO-IDIBELL), 08908 Barcelona, Spain; 5 Department of Environmental Health, Harvard School of Public Health, Boston, MA 02115 USA; 6 Department of Medicine, Massachusetts General Hospital, Boston, MA 02115 USA; 7 Department of Cancer Epidemiology, H. Lee Moffitt Cancer Center and Research Institute, Tampa, FL 33612 USA; 8 Markey Cancer Center, University of Kentucky, First Floor, 800 Rose Street, Lexington, KY 40508 USA; 9 National Institute of Occupational Health, 0033 Gydas vei 8, 0033 Oslo, Norway; 10 Laboratory Medicine Region Skåne, Department of Clinical Sciences Lund, Pathology, Lund University, Lund, Sweden; 11 Department of Clinical Sciences, Lund University, Malmö, Sweden; 12 Division of Public Health Sciences, Fred Hutchinson Cancer Research Center, Seattle, WA 98109 USA

**Keywords:** Genetic interactions, Machine learning, Genome-wide association study, Lung cancer

## Abstract

**Background:**

Genome-wide association studies (GWAS) have proven successful in predicting genetic risk of disease using single-locus models; however, identifying single nucleotide polymorphism (SNP) interactions at the genome-wide scale is limited due to computational and statistical challenges. We addressed the computational burden encountered when detecting SNP interactions for survival analysis, such as age of disease-onset. To confront this problem, we developed a novel algorithm, called the Efficient Survival Multifactor Dimensionality Reduction (ES-MDR) method, which used Martingale Residuals as the outcome parameter to estimate survival outcomes, and implemented the Quantitative Multifactor Dimensionality Reduction method to identify significant interactions associated with age of disease-onset.

**Methods:**

To demonstrate efficacy, we evaluated this method on two simulation data sets to estimate the type I error rate and power. Simulations showed that ES-MDR identified interactions using less computational workload and allowed for adjustment of covariates. We applied ES-MDR on the OncoArray-TRICL Consortium data with 14,935 cases and 12,787 controls for lung cancer (SNPs = 108,254) to search over all two-way interactions to identify genetic interactions associated with lung cancer age-of-onset. We tested the best model in an independent data set from the OncoArray-TRICL data.

**Results:**

Our experiment on the OncoArray-TRICL data identified many one-way and two-way models with a single-base deletion in the noncoding region of *BRCA1* (HR 1.24, *P* = 3.15 × 10^–15^), as the top marker to predict age of lung cancer onset.

**Conclusions:**

From the results of our extensive simulations and analysis of a large GWAS study, we demonstrated that our method is an efficient algorithm that identified genetic interactions to include in our models to predict survival outcomes.

## Background

A fundamental aim of studying human genetics is to predict disease risk from genomic data. Genome-wide association studies (GWAS) that used single-locus models by testing each single nucleotide polymorphism (SNP) for association with a phenotype, proved to be instrumental in identifying thousands of genetic variants associated with human traits and disorders [1–4]. However, most of the findings explained only a small proportion of the genetic effects on diseases and traits [1, 5]. The complex biological mechanisms and genetic architectures of diseases motivated researchers to not only study main additive effects of single genetic variations, but also interactions between multiple variants with non-additive effects to explain more of the heritability of complex diseases [6–10]. As the availability of large genome-wide genotype and next generation sequencing data continues to grow, detecting genetic interactions (i.e., SNP interactions) will become more feasible with increasing power to detect significant associations [11]. At the same time, epistasis detection faces computational and statistical challenges in analyzing high-dimensional data and in testing millions of interaction models from an exhaustive search in GWAS [6, 12]. The number of tests increases exponentially when analyzing higher orders of interactions, which require immense computing resources and processing time. Additionally, if the genotypic combinations that confer risk are nonadditive, finding the combinations of genotypes that increase risk can become a complex combinatorial challenge [7].

With the arrival of multi-dimensional and complicated genetic data sets, researchers have adapted to this growth by integrating machine learning methods to analyze complex genetic architectures. In genetic epidemiology, a popular series of methods were centered around a machine learning approach adapted to detect gene–gene interactions called the Multifactor Dimensionality Reduction (MDR) method. First introduced by Ritchie et al. (2001), MDR aimed at reducing high-dimensional genetic interacting loci to a one-dimensional binary variable that could be easily classified into high and low risk groups [7]. While MDR have successfully facilitated detection and characterization of multiple genetic loci, there were disadvantages to this algorithm that limited its use on diverse data structures such as survival data, which is often a primary outcome of interest in cancer research. Gui et al. [13, 14] have expanded on the MDR algorithm to different phenotypes, survival and continuous outcomes data. Survival MDR (Surv-MDR) extended the analysis of dichotomous traits in MDR to censored and time-to-event survival data using a log-rank test to classify sets of multi-loci combinations. This algorithm demonstrated proficiency in identifying genetic interactions associated with censored time-to-death or time-to-event data; however, it was more computationally demanding than MDR and it did not allow for covariate adjustments important for controlling confounding factors [13]. Quantitative MDR (QMDR) offered a computationally efficient algorithm to identify genetic interactions associated with a quantitative outcome, but it also did not allow for covariate adjustments such as age, gender, environmental toxins, and other confounding factors to accurately identify genetic association relations [14].

Currently, there are limited methods capable of identifying genome-wide genetic interactions efficiently with adjustment for covariates when studying age of disease-onset, such as a patient’s age at first diagnosis or recurrence of disease, for large-scale studies due to computational demands. It is important to have reliable estimates on the age of first diagnosis to understand the etiology of the disease and to tailor clinical practices, especially when determining the appropriate starting age for diagnostic screening, such as lung cancer screening [15]. In this study, we demonstrated how the Efficient Survival Multifactor Dimensionality Reduction (ES-MDR) method improved on the efficiency of Surv-MDR and allowed for adjustment of covariate effects to analyze large-scale survival and genetic data to analyze age of disease-onset in association with SNP interactions. Our method used Martingale Residuals as the estimated survival outcome with adjustment for confounding factors that provided an efficient and effective identification of genetic interactions associated with survival outcomes. We demonstrated the strength of the proposed method by designing two simulations to evaluate the 5% type I error threshold through an evaluation of the empirical null distribution and to analyze the predictive power of ES-MDR. To analyze the effectiveness of the ES-MDR method, we evaluated our approach using the genome-wide genotyped lung cancer OncoArray-TRICL (Transdisciplinary Research Into Cancer of the Lung) Consortium data to detect and characterize SNP interactions that were associated with lung cancer age-of-onset.

## Methods

In this section, we discuss how we improved the computational efficiency without reducing accuracy to develop the ES-MDR method when analyzing SNP interactions (i.e., joint effects of two SNPs) in association with age of disease-onset.

### Incorporating martingale residuals for age-of-onset survival analysis

ES-MDR improved the efficiency of Surv-MDR and applied the QMDR algorithm to analyze age of disease-onset in association with genetic interactions. Our novel ES-MDR approach used a combination of survival analysis and QMDR for continuous outcome analysis in two steps. In the first step, we started replacing event time and status with Martingale Residuals with covariate adjustment as a new continuous score. In the second step, we applied QMDR to efficiently categorize the genotype combinations into high-risk and low-risk groups. The best model was determined in the same way as QMDR, by using the cross-validated t-test statistic computed from a continuous variable attribute (e.g., Martingale Residuals) to determine the best interaction and overall model.

The novel algorithm for ES-MDR was performed as follows:

1. Selected K SNPs from all the SNPs in the data set and created a contingency table among every genotype combination of K SNPs.
2. For each multi-locus genotype combination cell, summed the Martingale Residuals between samples with and without each genotype combination.
3. Labeled cells “high-risk” if the sum of the Martingale Residuals was positive; otherwise negative Martingale Residuals were labeled “low-risk”.
4. Pooled all the high-risk labeled cells into one group and all the low-risk labeled cells into another group to create a new one-dimensional variable.

Using Martingale Residuals to determine high or low risk group for survival data analysis was comparable to using the log-rank test statistic in Surv-MDR, however, more efficiently when classifying genotype combinations. It can be shown that the sum of the Martingale Residuals is a good surrogate variable of the log-rank test statistics for the purpose of determining high/low risk groups for each genotype combination. Next, we compared the similarities of the equations for Martingale Residuals and the log-rank test statistic. The sign and magnitude of the Martingale Residuals were dependent on the association of SNPs and the hazard function in the following equation:

In this equation, δ_*i*_(*t*) denotes the number of observed events that occur at each survival time *t*. The number of expected events was calculated using the cox-proportional hazards model with *x* as the genetic factor and y as the adjusted covariate. The log-rank test statistic was defined as the following:

$$C = \frac{{\Sigma_{j = 1}^{J} \left( {O_{1j} - E_{1j} } \right)}}{{\sqrt {\Sigma_{j = 1}^{J} } V_{j} }}$$

Here, we show that Martingale Residuals is equivalent to the numerator of the log-rank test statistic. Therefore, the sum of the Martingale Residuals is equal to the log-rank test statistic when the variance is set to 1. This infers that using Martingale Residuals as a substitute for the log-rank test statistic in evaluating genomic combinations associated with survival outcomes could provide the same data reduction and categorization process as Surv-MDR.

### Evaluation through simulations

Our purpose of running a simulation study was to evaluate how well ES-MDR performed and how well it performed compared with Surv-MDR. To demonstrate the strength of the proposed method, two simulations were designed to evaluate the testing score’s null distribution to evaluate the type I error rate and to analyze power.

### Simulation I

The first simulation study was created to estimate the 5% type I error threshold by evaluating an empirical null distribution with independent non-interacting SNPs and quantitative outcome values. Here, we created sets of SNPs (m = 10, 20, 50) with additive coding and sample sizes (n = {200, 400, 800, 1600}) in the simulation data. For every combination of m and n, we simulated m SNPs with minor allele frequencies (MAF) drawn from the uniform distribution over the interval *U* (0.1, 0.5). Then we simulated n continuous outcomes from a standard normal distribution. The SNP and continuous outcome data were created independently to ensure that there were no associations between SNPs and the outcome. These steps were repeated to create 1000 null data sets for 24 different groups varied by the number of SNPs, sample size, and MAF. As a result, a total of 24,000 data sets were generated. Simulations were conducted in R 3.0.0 (Vienna, Austria). To determine whether the type I error rate was close to 5%, we analyzed the percentage of times that ES-MDR randomly identified two interacting SNPs from a null data set.

### Simulation II

The second simulation study was created to evaluate the power of ES-MDR with a data set that included quantitative outcome variables and a pair of functional interacting SNPs and 18 non-interacting SNPs. Surv-MDR was performed to evaluate whether ES-MDR was as effective as Surv-MDR in identifying functional SNPs.

The simulation data sets included different penetrance functions that described the probabilistic relationship between the quantitative outcome variable and functional SNPs generated with additive coding. We considered two different MAFs (0.2 and 0.4) and seven different broad-sense heritability statistics (0.01, 0.02, 0.05, 0.1, 0.2, 0.3, and 0.4) to create a total of 14 unique model combinations, where the two functional SNPs associated with the outcome were evenly distributed across the seven heritability statistics. To create a purely epistatic model, each of the 14 unique models had one or the other functional SNP (MAF 0.2 or 0.4) with no main effects. The 14 allele-heritability frequency combinations were replicated five times to generate 70 models with varying sample sizes that included size (n) = {400, 800, 1600}.

Assuming SNP1 and SNP2 were the two functional SNPs. Let *f*_ij_ be an element from the *i*th row and *j*th column of a penetrance function. We generated the binary variable from a Bernoulli distribution with the following:

$$P\,\left( {{\text{high}}\,{\text{risk}}|{\text{SNP}}1 \, = i,\,{\text{SNP}}2 = j} \right) = f_{ij}$$

We randomly selected 200 high-risk subjects and 200 low-risk subjects from each of the 70 probabilistic models to create one simulated data set. We repeated this simulation 100 times to obtain at total of 7,000 data sets.To generate the survival time, we used the Cox-proportional hazards (Cox ph) model:

$${\text{h}}\left( {t|x} \right) = {\text{h}}_{0} \left( t \right)\exp\left( {{\beta}x} \right)$$

In this equation, h_0_(*t*) is the baseline hazard function with a Weibull distribution using the shape parameter of 5 and the scale parameter of 2. The *x* is the genetic factor fixed at value 1 for high risk patients and 0 for low risk patients. ß represents the effect size or the log hazard ratio for a one-unit increase in *x* (all other covariates held constant). The censoring fractions were sampled from the uniform distribution over the interval *U* (0,4) from the Bernoulli distribution, resulting in 40% censoring. Finally, we merged survival time and censoring status with the SNP data.

We used Martingale Residuals in our novel ES-MDR method to classify each multi-locus genotype combination into high-risk and low-risk groups. The Martingale Residual is the stochastic component and in residual form gives the following:

$${\text{M}}\left( {t|x} \right) = \delta \left( t \right) - h_{0} \left( t \right)\exp\left( {{\beta}x} \right)$$

In this example, δ(*t*) denotes the number of expected events that occurred at each survival time *t*. Assuming a null model with no target effects (ß = 0), this residual is the difference between the observed events and expected number of events. The sign and magnitude of the Martingale Residuals are dependent on the association of SNPs and the hazard rate function. Each individual genotype with a positive Martingale Residual (i.e., greater than or equal to 0) was classified as high-risk. Otherwise, a negative Martingale Residual was classified as low-risk. For every multi-locus genotype combination of SNPs, we computed the sum of the Martingale Residuals to obtain a new variable that could be used to classify into the high-risk or low-risk group.

To estimate the power of the proposed method, we ran ES-MDR on each of the 7000 data sets and searched for the best model over all possible one- (i.e., single-locus), two- (i.e., two interacting loci), and three-way (i.e., three interacting loci) interaction models, using the T-statistic testing score. We also used the 95th percentile of the testing score from the null models as a threshold to guard against any non-significant findings. The power was estimated as the percentage of time ES-MDR correctly included the two functional interacting SNPs in the best model out of each set of 7000 data sets. This significant threshold for the results was at the 0.05 level. For comparison, we ran Surv-MDR on the simulated data to define its power. Training and testing scores for ES-MDR were analyzed using two-fold cross-validation. The rational for using two-fold cross-validation [16] was that there would be no overlap between training sets and that all the predicted values were independent of each other. The best model was selected with the smallest prediction error and largest consistency in including the two functional interacting SNPs.

### OncoArray-TRICL genotyping and quality control

A total of 533,631 SNPs from 57,775 individuals in the OncoArray-TRICL Consortium population-based study, selected from 29 studies across North America and Europe, as well as Asia, were genotyped using the Illumina OncoArray-500K BeadChip Platform, which included the genome-wide backbone and select loci known to be associated with cancer phenotypes. To facilitate efficient genotyping and minimize variability that might arise from genotyping at multiple sites, genotyping was conducted at the following five institutions: the Center for Inherited Disease Research, the Beijing Genome Institute, the Helmholtz Zentrum München, Copenhagen University Hospital, and the University of Cambridge. Quality control steps described previously were followed for this OncoArray-TRICL data set [17]. The following participants were excluded from the current study: participants who lacked lung cancer status (did not participate in the lung cancer studies), smoking status, and age and gender information at diagnosis, participants who were close relatives (second degree relatives or closer), duplicate individuals, with non-European ancestry, with low-quality extracted DNA, with low call-rate for genotype data, and participants who did not pass other quality control measures. As a result, a total of 14,935 lung cancer cases and 12,787 controls remained in the current study. We restricted SNP filtering to a minimum to include more SNPs for analysis. We included SNPs with MAF ≥ 0.01 and SNPs with 50% and above genotyping rate.

### OncoArray-TRICL data analysis

We applied ES-MDR to the OncoArray-TRICL Consortium population-based study to identify genetic interactions in association with lung cancer age-of-onset. The OncoArray-TRICL Consortium is a collaboration among world leaders to investigate common causes of cancer susceptibility and progression [17]. Lung cancer cases and controls were genotyped using the OncoArray genotyping array known to tag cancer traits and susceptibility loci in addition to the GWAS backbone; this array consisted of approximately 533,000 tagged SNPs. We identified 27,722 participants (14,935 lung cancer cases and 12,787 healthy controls) aged 15–96 years of European ancestry. All participants provided informed consent and each study site obtained approval from their ethics committee. In this analysis, lung cancer age-of-onset, cases (event at diagnosis age), controls (censored at interview age), and a covariate (smoking status) constituted the survival outcome data that were substituted by Martingale Residuals. We randomly sampled 2/3 of the data into a training set and 1/3 as the testing set. We applied our novel ES-MDR method to perform an exhaustive one-way and two-way model search. We used PLINK as a pre-filtering step to identify uncorrelated and independent SNPs. SNPs that were in linkage disequilibrium were removed, using a stringent correlation threshold of 0.1. After this filtering step, 108,254 SNPs remained. We searched over all one-way and two-way interactions in the training set to identify models consistently selected with the largest training score determined by two-fold cross-validation and we analysed the prediction error of the chosen top 10 models in the testing set. In our real data analysis, we also considered joint detection of the two SNPs with main effects to be successful detection of the functional interaction model. We performed a 10,000-fold permutation test to evaluate the significance of chosen models.

To build a predictive model that combined the strength of both one-way and two-way models, we took all the SNPs involved in the top 1000 one-way models and all the SNPs from the top 1000 two-way interactions models and applied a penalized Cox regression method to filter and select the best predictive models to evaluate genetic factors associated with age of lung cancer onset. We ranked the test scores from highest to lowest and picked the top SNPs that best predicted lung cancer onset.

To construct predictive models linking SNPs to censored survival data, we used the least absolute shrinkage and selection operator (Lasso) penalized estimation for the Cox regression model to select top SNPs that were relevant to patients’ ages of lung cancer onset to create a prediction model with a parsimonious set of SNPs that could provide good prediction accuracy [18]. The Lasso procedure is a popular method for variable selection when the number of samples is significantly less than the number of predictor variables in the prediction model [19]. Briefly, Lasso is similar to the forward stepwise method in that it provides coefficient shrinkage as well as variable selection by driving nonsignificant coefficients in a regression model to zero [19]. Therefore, Lasso is a valuable tool to filter SNPs that are not associated with the outcome or highly correlated with other SNPs, especially in situations when the sample size is smaller than the number of SNP predictors.

Survival plots were generated using the Kaplan–Meier (KM) method to visualize the differences in age of lung cancer onset between high-risk and low-risk groups based on top identified SNPs associated with lung cancer risk. To adjust for additional factors related to patient survival, the Cox ph regression model included adjustment for smoking status as a covariate in the model.

To assess the performance of our model in predicting lung cancer onset at different age intervals, we applied time-dependent receiver operating characteristic (ROC) curve and area under the curve (AUC) to evaluate the predictive performance of the best models, previously introduced by Heagerty et al. [20]. In our study, with a given score function *f*(*X*), the time-dependent sensitivity and specificity functions were defined as follows:

$$\begin{aligned} & sensitivity\left( {c,t|f\left( X \right)} \right) = \Pr \left\{ {f\left( X \right) > c|\delta \left( t \right) = 1} \right\}, \\ & specificity\left( {c,t|f\left( X \right)} \right) = \Pr \left\{ {f\left( X \right) \le c|\delta \left( t \right) = 0} \right\}, \\ \end{aligned}$$

We defined the corresponding ROC(*t*|*f*(*X*)) curve for any time *t* as the plot of *sensitivity*(*c*, *t*|*f*(*X*)) versus 1—*specificity*(*c*, *t*|*f*(*X*)) with the cut-off point *c* varying. The AUC is the area under the ROC(*t*|*f*(*X*)) curve, which was denoted as AUC(*t*|*f*(*X*)) [18]. Here, the *δ*(*t*) is the event indicator at time *t*. In this study, a larger AUC at time *t* based on the score function *f*(*X*) indicated better predictability of time-to-event at time *t* as measured by sensitivity and specificity evaluated at time *t*.

## Results

### Assessing type 1 error in simulation I

In the first simulation, we determined whether the type I error rate was close to the expected value when there were no SNP interaction effects. Assuming a data set that included 20 non-interacting SNPs and a total sample size of 400, we expected the type I error rate to be 0.05.

**Fig. 1:**
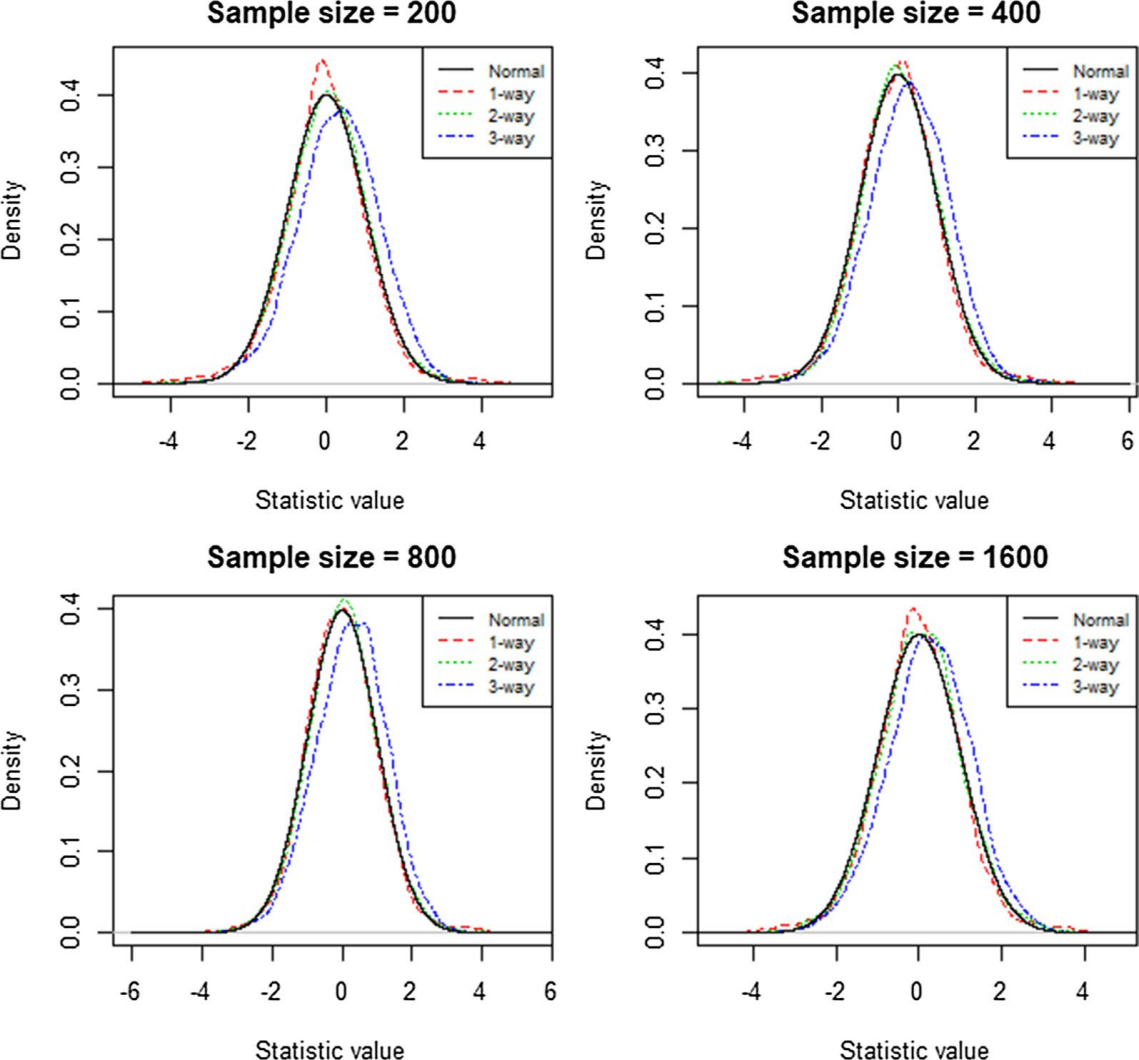
Empirical Distribution of the two-fold cross-validated testing scores. Each graph for sample sizes 200, 400, 800 and 1600 displays four curves that represent the testing score distributions for a one-, two-, and three-way model and the standard normal distribution

In Fig. 1, the null distributions for the one- and two-way models followed the normal distribution quite closely, whereas the three-way model displayed a slight right skew. Nevertheless, the upper right tail regions almost perfectly overlapped with the upper right tail of the normal distribution for all three interaction models. This showed that the use of the 95th quantile of the empirical distribution as a threshold to remove false positives was suitable. This would greatly reduce the computing time by comparing testing scores with the prior calculated empirical distribution [14]. In Table 1, we used the 95th quantile of data sets with 400 samples to estimate the type 1 error rate. The estimated rate for type I error was tightly distributed around 5% with a range from 4.5 to 5.6% for the one-way and two-way models. The estimated error rate for the three-way model was greater with a range from 7.5 to 9.7%; however, it also exhibited a trend towards 5% with increasing sample size. Based on the results in Table 1, simulation I revealed that with every two-fold increase in the sample size, there was an average 0.6% decrease in error rate for the three-way model. As a result, we expected that the type I error rate would converge to 5% with sample sizes greater than approximately 12,800.

**Table 1:** Estimated type I error rate in simulation i using the 95th quantile of the standard normal distribution

m^a^ = 20	n^a^ = 200 (%)	n^a^ = 400 (%)	n^a^ = 800 (%)	n^a^ = 1600 (%)	n^a^ = 3200 (%)
1-way	4.5	4.7	4.9	4.8	5.0
2-way	5.3	5.6	5.2	5.4	5.4
3-way	9.7	8.7	8.1	7.5	7.8

^a^m, number of SNPs; n, sample size

### Assessing power and speed in simulation II

In the second simulation, we estimated the power of ES-MDR with a data set that included quantitative outcome variables and a pair of functional interacting SNPs and 18 non-interacting SNPs. We determined whether the power of ES-MDR was comparable to Surv-MDR in identifying the two functional SNPs. We counted the number of times that the functional SNP pair was correctly identified and divided that number by the total number of data sets (500 for this simulation) to get the estimated success rate.

**Fig. 2:**
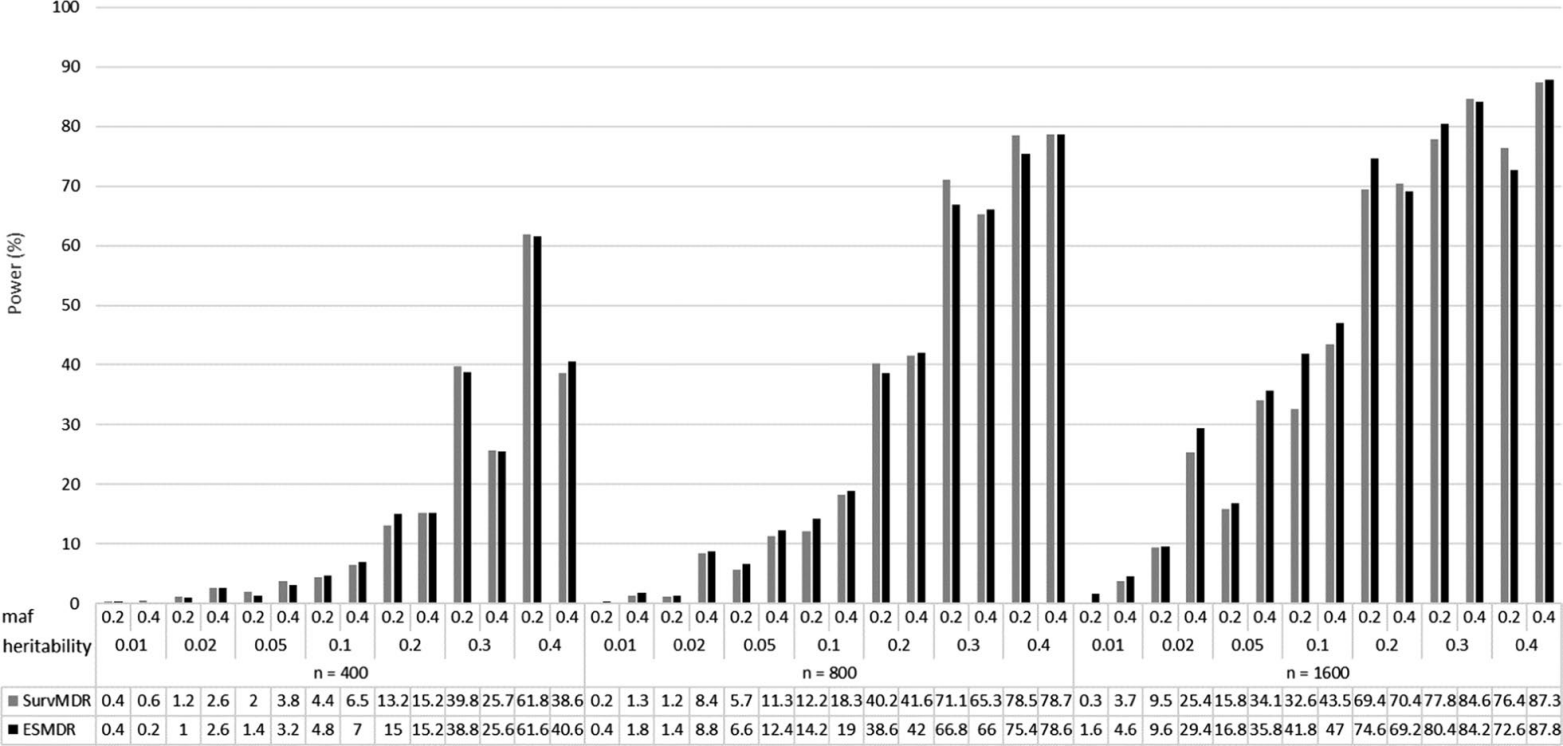
Simulation II Power Comparison between ES-MDR and Surv-MDR – Stringent Model. The stringent model included two interacting SNPs (i.e., SNP1 and SNP2) and used n—sample sizes (e.g., 400, 800, 1600), maf—minor allele frequencies (e.g., 0.2, 0.4) and heritability statistics (e.g., 0.01, 0.02, 0.05, 0.1, 0.2, 0.3, 0.4)

Figure 2 presents a comparison of the power to identify only the two (i.e., stringent model) interacting SNPs (SNP1 and SNP2) for ES-MDR and Surv-MDR on simulated data. Table 2 displays the percent change in power to detect only the two functional interacting SNPs between ES-MDR and Surv-MDR. Overall, ES-MDR performed better than Surv-MDR for larger sample sizes. In addition, both ES-MDR and Surv-MDR demonstrated increasing power to detect functional SNPs with increasing heritability frequencies.

**Table 2:** Percent change in power between ES-MDR and Surv-MDR

Heritability	0.01 (%)	0.02 (%)	0.05 (%)	0.10 (%)	0.20 (%)	0.30 (%)	0.40 (%)
n^a^ = 400							
maf^a^							
0.2	0.0	− 16.7	− 30.0	9.1	13.6	− 2.5	− 0.3
0.4	− 66.7	0.0	− 15.8	7.7	0.0	− 0.4	5.2
n^a^ = 800							
maf^a^							
0.2	100.0	16.7	15.8	16.4	− 4.0	− 6.0	− 3.9
0.4	38.5	4.8	9.7	3.8	1.0	1.1	− 0.1
n^a^ = 1600							
maf^a^							
0.2	433.3	1.1	6.3	28.2	7.5	3.3	− 5.0
0.4	24.3	15.7	5.0	8.0	− 1.7	− 0.5	0.6

^a^n, sample size; maf, minor allele frequency;

^b^% change calculator = ((ES-MDR − Surv-MDR)/Surv-MDR) × 100%

**Fig. 3:**
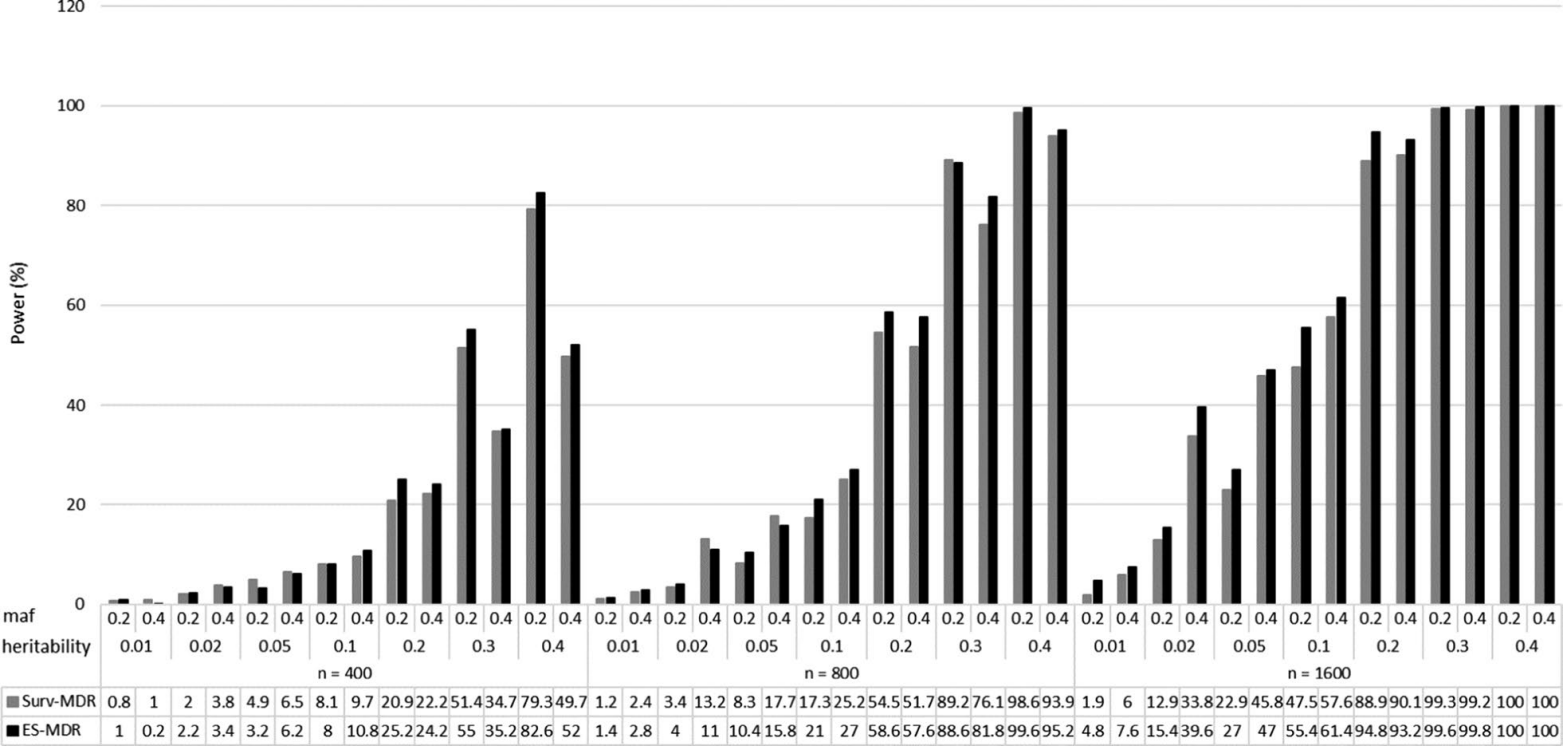
Simulation II Power Comparison between ES-MDR and Surv-MDR—Flexible Model. The flexible model included two interacting SNPs (i.e., SNP1 and SNP2) plus a third SNP3 and used n—sample sizes (e.g., 400, 800, 1600), maf—minor allele frequencies (e.g., 0.2, 0.4) and heritability statistics (e.g., 0.01, 0.02, 0.05, 0.1, 0.2, 0.3, 0.4)

Figure 3 displays a comparison in power to identify the two interacting SNPs (SNP1 and SNP2) plus an additional SNP (i.e., flexible model) between ES-MDR and Surv-MDR. Table 3 shows the percent change in power to detect the two interacting SNPs plus an additional SNP. Here, we also demonstrated that ES-MDR had greater power compared to Surv-MDR. Again, ES-MDR performed better than Surv-MDR with larger sample sizes.

**Table 3:** Percent change in power between ES-MDR and Surv-MDR

Heritability	0.01 (%)	0.02 (%)	0.05 (%)	0.10 (%)	0.20 (%)	0.30 (%)	0.40 (%)
n^a^ = 400							
maf^a^							
0.2	25.0	10.0	− 34.7	− 1.2	20.6	7.0	4.2
0.4	− 80.0	− 10.5	− 4.6	11.3	9.0	1.4	4.6
n^a^ = 800							
maf^a^							
0.2	16.7	17.6	25.3	21.4	7.5	− 0.7	1.0
0.4	16.7	− 16.7	− 10.7	7.1	11.4	7.5	1.4
n^a^ = 1600							
maf^a^							
0.2	152.6	19.4	17.9	16.6	6.6	0.3	0.0
0.4	26.7	17.2	2.6	6.6	3.4	0.6	0.0

^a^n, sample size; maf, minor allele frequency;

^b^% change calculator = (ES-MDR − Surv-MDR)/Surv-MDR × 100%

We compared the computing time between ES-MDR and Surv-MDR for 100 simulated data sets, for one-, two-, and three-way interactions, and with ten-fold cross-validation. The computing time for Surv-MDR was 734.5 min versus 2.25 min for ES-MDR, both of which were run on 1 node in the high-performance computing cluster called Discovery with AMD 3.1 Ghz CPU and 64 GB of memory. Discovery uses a Linux RedHat 6.7 operating system and is comprised of 160 computing nodes (3000 + cores), 12.5 TB of memory, and is available to the Dartmouth research community.

### Application to OncoArray-TRICL data set

The main goal was to identify SNPs with main effects and SNP interactions that were associated with lung cancer susceptibility at different ages of disease onset. Using a population-based study, we applied ES-MDR on the OncoArray-TRICL Consortium data with 14,935 cases and 12,787 controls for lung cancer to search over all one-way and two-way interactions to identify genetic interactions in relation to lung cancer age-of-onset. For this study, we included 533,631 genotyped variants and removed SNPs in linkage disequilibrium (LD) > 0.1 (n = 108,254 SNPs).

**Fig. 4:**
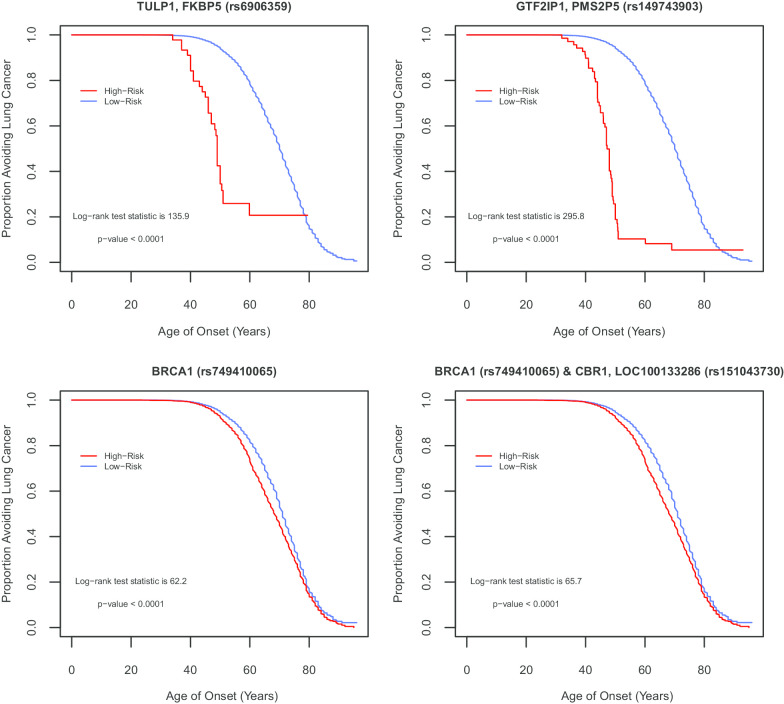
Differences between High Risk (red curve) and Low Risk (blue curve) groups in relation to SNPs. Kaplan–Meier plots displays the difference between individuals categorized in High Risk vs. Low Risk groups by genetic variation in top one-way SNPs identified from the testing set, TULP1/FKBP5 (rs6906359), GTF2IP1/PMS2P5 (rs149743903), and BRCA1 (rs749410065) (NC_000017.10:g.41196821delT), and in a top two-way interacting SNPs from the testing set, BRCA1 (rs749410065) (NC_000017.10:g.41196821delT) & CBR1, LOC100133286 (rs151043730)

**Fig. 5:**
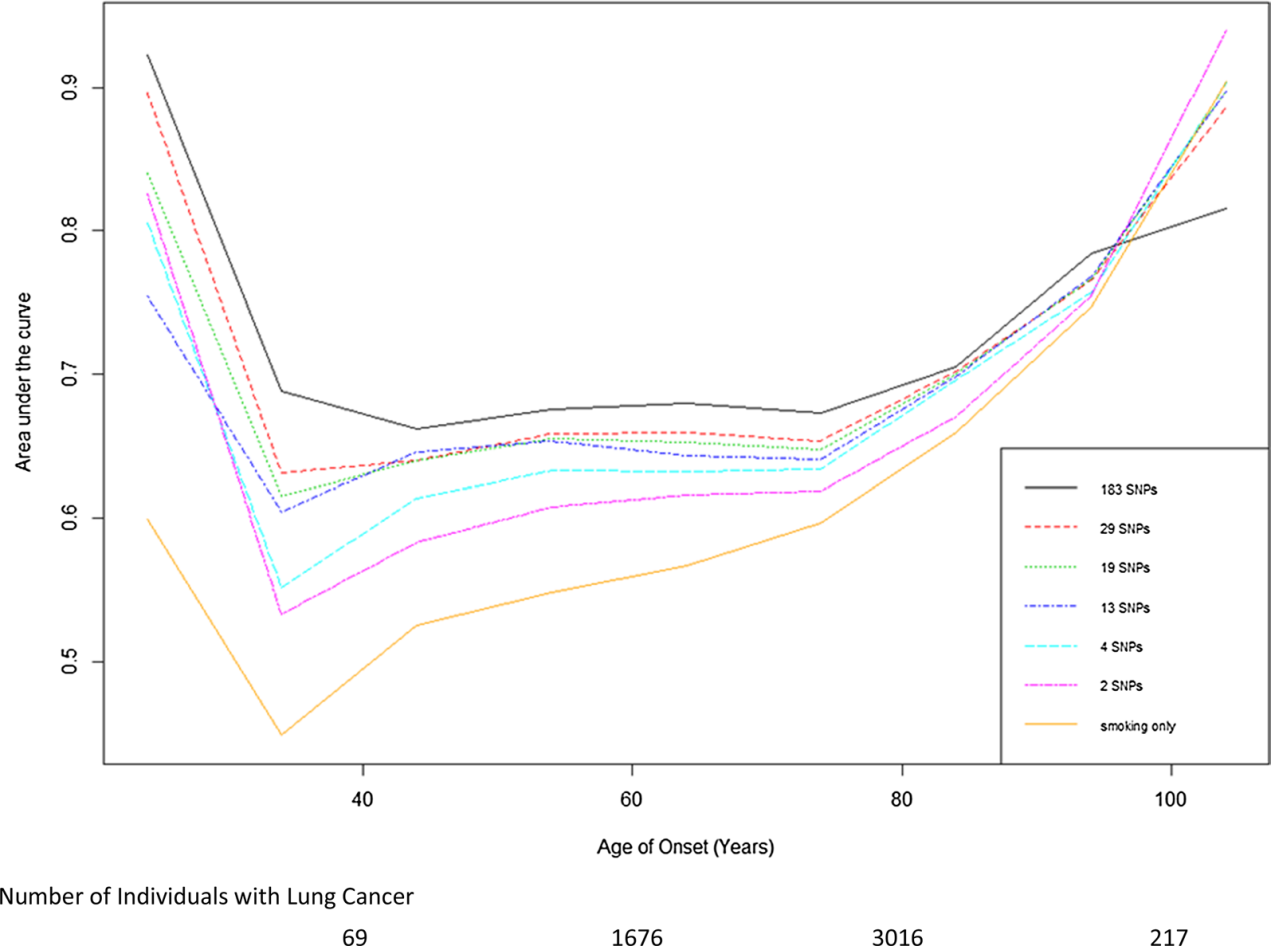
Plots of Area under the curve (AUC). Each line corresponds to a model. Models were smoking only and smoking plus 2 to 183 SNPs. Here we compared the AUCs between the smoking only model and smoking plus SNPs models for predicting lung cancer age-of-onset ranging from 20 to < 100 years. The number of individuals diagnosed with lung cancer at < 40 years, 40 to < 60 years, 60 to < 80 years, and 80 to < 100 years is shown below the figure

Table 4 lists the top 10 one-way test results generated by ES-MDR and cross-validation. Using ES-MDR, highly significant SNPs were identified in association with lung cancer age-of-onset. Table 5 displays the top 10 two-way interactions identified by ES-MDR that were associated with lung cancer age-of-onset. Due to the observed inflation of the type 1 error rate for 3-way interactions in the simulation study, a 3-way interaction was not evaluated in the OncoArray-TRICL data analysis. For Table 6, we combined SNPs from the top 1000 one-way loci and the top 1000 two-way interactions, ranked the SNP scores from highest to lowest, and applied the Lasso Cox regression method to filter and select the best genetic factors that predicted age of lung cancer onset. Table 6 exhibits the top 10 significant SNPs selected by Lasso Cox regression. To visualize the difference in age of lung cancer onset between the high risk and low risk groups, Fig. 4 illustrates the contrast using the KM) survival curve. KM curves for top one-way SNPs in the intronic region of *TULP1*, *FKBP5* (rs6906359), in-between genes *GTF2IP1*, *PMS2P5* (rs149743903), and in the deletion of a noncoding region of *BRCA1* (rs749410065) (NC_000017.10:g.41196821delT per Human Genome Variation Society nomenclature), and for a top two-way interacting SNPs in gene regions of *BRCA1* (rs749410065) and *CBR1, LOC100133286* (rs151043730) displayed a clear separation of curves between the high- and low-risk groups. This demonstrated the efficacy of ES-MDR using Martingale Residuals to differentiate high risk and low risk groups based on genotype variation when evaluating lung cancer age-of-onset. We continued our analysis with a comparison of smoking only and smoking plus SNP models to determine the best performance in predicting lung cancer onset at different ages. We used a common graphical plot called the area under the receiver operating characteristic (ROC) curve, also known as AUC, to measure the performance of our models to discriminate the best parameters at predicting lung cancer onset at different ages based on accuracy. In Fig. 5, the x-axis corresponds to the age of lung cancer onset, starting from 15 to > 80 years, and the y-axis indicates the AUC, ranging from 0.4 to 1. We examined the predictive performance of 7 different models with various tuning parameters identified from Cox Lasso regression, such as smoking only and smoking plus 2 SNPs, 4 SNPs, 13 SNPs, 19 SNPs, 29 SNPs, and 183 SNPs. This figure shows the average of the estimated AUCs over the OncoArray-TRICL data using the predictive scores from the independent left-out test data set. The plot displays good predictive performances of models generated using ES-MDR. The AUC for models with more SNPs lies between 0.6 and 0.7 and continues to increase at later ages of onset. There is a noticeable decrease in AUC for ages 40 and below. This could be due to the limited number of lung cancer cases identified for individuals below the age of 40, which indicated that the models might not be appropriate to predict lung cancer diagnoses at 40 years and younger. The AUC of both smoking only and smoking with SNPs increased with age from age 40 and older. However, the AUC, depending on the number of SNPs in the models, differed by age. The model with the largest number of SNPs plus smoking performed the best at AUC 0.68 between ages 40 and 80 of onset compared to the smoking-only model with an AUC of 0.55. There was a noticeable trend where incremental additions of SNPs in the model increased the AUC for age-of-onset between 40 and 80 + . On the other hand, the AUC for smoking-only and smoking plus fewer SNP models (e.g., 2 and 4) displayed the opposite trend where it increased around 90 + years of age.

**Table 4:** Top-one-way models identified by ES-MDR in OncoArray-TRICL data

Nearest Gene(s)	Chr^a^	SNP (GRCh37/hg19)	Position (bp^a^) GRCh37/hg19	Gene Region	Alleles (Major/Minor)	MAF^a^ (1000 Genomes)	Log-rank Test score	OncoArray-genotyped (HR^a^)	Permutation *P* value
*LINC00708, LOC105755953*	10	rs12358150	8735744	Intergenic	C/T	0.26	340.90	20.51	< 0.0001
*GTF2IP1, PMS2P5*	7	rs149743903	74711828	Intergenic	T/C	NA^a^	295.80	6.39	< 0.0001
*PPP2R2B, STK32A*	5	rs76601208	146581977	Intron	C/T	0.002	180.30	22.83	< 0.0001
*KLF5, LINC00392*	13	rs138428539	73736950	Intergenic	T/C,G	0.01	145.10	6.46	< 0.0001
*TULP1, FKBP5*	6	rs6906359	35528378	Intron	C/T	0.10	135.90	5.76	< 0.0001
*UTP23, RAD21*	8	rs10105870	117807762	Intergenic	G/A	0.15	113.00	27.64	< 0.0001
*VPS8*	3	rs112047443	184701960	Intron	A/T	NA^a^	93.30	19.57	< 0.0001
*BRCA1*	17	rs749410065	41196821	delT^a^	-/T	NA^a^	62.20	1.24	< 0.0001
*ATR*	3	rs529613417	142285472	Intron	A/T	0.001	57.00	5.48	< 0.0001
*B3GNT2, TMEM17*	2	rs11526118	62647317	Intron	G/A	0.16	46.00	23.01	0.0033

^a^Chr, chromosome; bp, base pair; MA, minor allele frequency; detT, NC_000017.10:g.41196821delT; NA, not available; HR, hazard ratio

**Table 5:** Top two-way models identified by ES-MDR in OncoArray-TRICL data

Gene(s) 1	SNP 1 (GRCh37/hg19)	Gene Region 1	Alleles (Major/Minor)	MAF^a^ (1000 Genomes)	Gene(s) 2	SNP 2 (GRCh37/hg19)	Gene Region 2	Alleles (Major/Minor)	MAF (1000 Genomes)	ES-MDR Test Score	OncoArray-genotyped (HR^a^)	Log-rank test statistic	Permutation *P* value
*BRCA1*	rs749410065	delT^a^	–/T	NA^a^	*CBR1, LOC100133286*	rs151043730	missense, ncRNA^a^	G/A	0.001	13.77	1.24	65.74	< 0.0001
*BRCA1*	rs749410065	delT^a^	–/T	NA^a^	*NAPG*	rs3865365	near 5′ end of genes	G/A	0.06	13.43	1.23	61.74	< 0.0001
*C6orf10*	rs16870005	Intron	C/T	0.01	*BRCA1*	rs749410065	delT^a^	–/T	NA^a^	13.42	1.25	67.66	< 0.0001
*C1orf21, LOC107985236*	rs7535067	Intron, 3′ UTR^a^	C/A,T	0.19	*BRCA1*	rs749410065	delT^a^	–/T	NA^a^	13.39	1.24	61.92	< 0.0001
*PSMB9, LOC100294145*	rs57092860	Intergenic	T/C,G	0.01	*BRCA1*	rs749410065	delT^a^	–/T	NA^a^	13.37	1.25	67.82	< 0.0001
*CTNND2*	rs7732411	Intron	T/C	0.08	*BRCA1*	rs749410065	delT^a^	–/T	NA^a^	13.32	1.25	67.53	< 0.0001
*TRAM2-AS1, LOC730101*	rs182398206	Intergenic	A/G	0.01	*BRCA1*	rs749410065	delT^a^	–/T	NA^a^	13.30	1.25	70.95	< 0.0001
*MIR4417, MIR4689*	rs6698924	Intergenic	A/C	0.02	*BRCA1*	rs749410065	delT^a^	–/T	NA^a^	13.29	1.25	68.74	< 0.0001
*BRCA1*	rs749410065	delT^a^	–/T	NA^a^	*TFAP2C, BMP7*	rs186132350	Intergenic	T/A	0.01	13.29	1.25	69.79	< 0.0001
*HRAT92, PRKAR1B*	rs142263110	Intergenic	G/A	0.002	*BRCA1*	rs749410065	delT^a^	–/T	NA^a^	13.28	1.25	69.36	< 0.0001

^a^UTR, untranslated region; delT^a^, NC_000017.10:g.41196821delT; ncRNA, noncoding transcript variant; HR, hazard ratio; NA, not available

**Table 6:** Top SNPs selected by cox lasso regression in OncoArray-TRICL data

Nearest Gene(s)	Chr^a^	SNP (GRCh37/hg19)	Position (bp^a^) (GRCh37/hg19)	Gene region	Alleles (Major/Minor)	MAF^a^ (1000 Genomes)	Test score	OncoArray-genotyped (HR^a^)	Permutation *P* value
*BRCA1*	17	rs749410065	41196821	delT^a^	–/T	NA^a^	62.20	1.24	< 0.0001
*GTF2IP1, PMS2P5*	7	rs149743903	35528378	Intergenic	C/G	NA^a^	295.80	6.39	< 0.0001
*LOC102467079, TOX3*	16	rs117142114	52328666	Intergenic	T/C	0.02	31.00	1.29	< 0.0001
*HYKK*	15	rs9788721	78802869	Intron	C/T	0.31	86.30	1.41	< 0.0001
*MIR3925, PANDAR*	6	rs7753169	36614326	Intergenic	A/C	0.36	27.90	1.15	< 0.0001
*CHRNA5*	15	rs16969968	78882925	missense	G/A	0.15	86.80	1.41	< 0.0001
*KLF5, LINC00392*	13	rs138428539	73736950	Intergenic	T/C,G	0.01	145.10	6.46	< 0.0001
*TULP1, FKBP5*	6	rs6906359	35528378	Intron	C/T	0.10	135.90	5.76	< 0.0001
*CHRNA5*	15	rs951266	78878541	Intron	G/A	0.16	88.50	1.41	< 0.0001
*FAM114A1*	4	rs1873195	38891173	Intron	C/T	0.20	8.90	0.87	0.0119

^a^Chr, chromosome; NA, not available; bp, base pair; delT^a^ = NC_000017.10:g.41196821delT; MAF, minor allele frequency; HR, hazard ratio

## Discussion

In this study, we present a novel algorithm to identify genetic interactions associated with the age-of-onset for lung cancer. We demonstrated in two simulation studies that our ES-MDR method was properly controlled for at the 5% type I error rate under the null distribution and improved power to detect causal SNPs. We identified new loci that were biologically plausible for lung cancer onset using the large OncoArray-TRICL data with 27,722 individuals. There are two unique contributions from this study. First, we offer a more computationally efficient algorithm, ES-MDR, a method that analyses survival data by using Martingale residuals in place of survival outcome data. Second, ES-MDR includes the ability to adjust for covariates, such as smoking status, a necessary step to control for confounding factors, whereas existing methods, used for survival analysis such as Surv-MDR, are unable to provide.

Using the MDR method to reduce the size of multiple dimensions to a single dimension to identify multi-locus genetic interactions in high-dimensional genomic data sets has been a well-established approach. Richie et al. (2001) first introduced MDR, a non-parametric (i.e., no parameters are estimated) and genetic model-free (i.e., no genetic model is assumed) model, that condensed multiple genetic loci into a single variable in order to categorize genotypes into two groups [7]. The goal was to group genotypes into high-risk and low-risk categories associated with and without disease outcomes, respectively. However, MDR was restricted by its inability to analyze different outcome variables other than binary variables and it did not allow for the adjustment of confounding factors that was critical in preventing false association analyses. Therefore, an extension of the traditional MDR method was developed to analyze censored survival data, called Survival MDR or Surv-MDR.

Like the original MDR algorithm, Surv-MDR is a non-parametric and genetic model-free method proposed by Gui et al. (2011), and it was developed to allow for the analysis of time-to-event data, such as patient survival time or time to disease relapse [13]. Surv-MDR used the log-rank test statistic to compare survival times between samples with and without the multi-locus risk genotype combination and classified them into high and low risk groups [13]. Surv-MDR also used cross-validation to identify the optimal set of K SNPs and overall best model. While Surv-MDR was successful in identifying SNP interactions associated with time-to-event outcomes, it was more computationally demanding than MDR and the inability to adjust for covariates persisted. Consequently, the MDR method was optimized further to develop the Quantitative MDR (QMDR) method to address the slow-to-compute algorithm challenge [14].

QMDR optimized the MDR algorithm by offering a computationally efficient way to analyze quantitative or continuous trait outcomes. QMDR compared the mean value of each multi-locus genotype to the overall mean and labeled each genotype combination as “high-risk” or “low-risk”. Cross-validation was also implemented in QMDR to identify the optimal set of K SNPs and overall best model. For each K-way interaction, the steps used for a k-fold cross-validation were similar to the Surv-MDR method except for the step to identify the best K-way interaction. In this case, the largest T-test statistic was used instead of the square of the log-rank statistic when identifying the best interaction model. Inspired by the computational capabilities of QMDR to analyze quantitative outcomes associated with genetic variations, we leveraged this method’s straightforward computing efficiency to evaluate survival outcome data for time-to-event analysis.

Our approach transformed survival data (e.g., time and event status) into a single variable, Martingale Residuals, to use as a surrogate for time-to-disease and disease status, with application of QMDR for rapid processing of genotype combinations into high and low risk groups. We were able to identify thousands of significant one-way and two-way models using ES-MDR and cross-validation when applied to the lung cancer OncoArray-TRICL data set. We were unable to compare the results of ES-MDR and Surv-MDR, both because Surv-MDR would have taken an extensive amount of time (e.g., greater than 4 months) to conduct a genome-wide genetic interaction analysis using the large OncoArray-TRICL data set, and because the current Surv-MDR algorithm would not allow for adjustment of confounding factors such as smoking status.

When searching for SNP interactions using real data, we chose a two-fold cross-validation instead of a ten-fold cross-validation to evaluate the optimal one-way and two-way interaction models as described previously [14]. From the central limit theorem, assuming a sufficiently large sample size (n > 50) from a population with a finite level of variance, the mean of all samples from the same population would be approximately equal to the mean of the population. Therefore, we expected the testing scores with 400 samples from our simulation study to follow a standard normal distribution. However, Gui et al. (2013) displayed a slight right skew with a standard deviation of 1.6 in their empirical distributions that was due to extra variation introduced by overlapping training sets in their ten-fold cross-validation method [14, 21]. Furthermore, two-fold cross-validation had been advocated to perform hypothesis testing where the training folds were mutually independent with no overlap [21]. Consequently, we evaluated the optimal one-way and two-way interaction models and the overall best model using two-fold cross-validation.

We explored prediction models that included SNPs that could be used to forecast lung cancer onset. Figure 5 lays out the AUC estimates for each model. The AUC peaked around age-of-onset less than 30 and greater than 90 years old. This may be due to the limited number of lung cancer cases (e.g., less than 10 cases) at younger and older ages. In general, based on AUC averages, age of lung cancer onset was strongly influenced by genetic variants, with increasing numbers of SNPs contributing to better AUC estimates. The plateauing of AUC averages for the 40–80 years old range revealed good estimates for age of onset for all models, which was likely due to the larger sample size for evaluation. Another plausible explanation for the high AUC for early and late age of onset was the likelihood that those cases contained the same combinations of risk SNPs in the models. The identified top SNPs with high AUC for age of onset were not only associated with early lung cancer cases, but they potentially could also contribute to late age of onset cases. The 2 SNP and 4 SNP models had strong associations with lung cancer cases and therefore, were responsible for high AUC averages for early and late age of onset of lung cancer. For the smoking only model, it played less of a role for early lung cancer onset because the adverse effects from smoking could require more time to develop. Over time the effects from smoking could be the main driver for late age lung cancer cases, which could explain why genetic factors do not seem to greatly effect cancer onset in later years. This interpretation could make biological sense since the effect of smoking over a longer time period could have compounding effects on cancer development. Conversely, cancer development due to genetics might appear at earlier rather than later years.

### Limitations

While our novel ES-MDR overcame some of the limitations described in previous methods used to evaluate genetic interactions, it was not without some of its own disadvantages. When analyzing survival data, the method did not directly evaluate survival variables such as time and event status. As a result, when using Martingale Residuals instead of specific survival outcomes data, we might be missing some important information that was needed to identify associations between SNP interactions and survival outcomes. Our QQ plot analysis from real data indicated a strong departure from the null distribution which indicated that there might exist a systematic bias. This result could be due to a combination of the large sample size and continuous outcome. As a result, we used permutation tests to evaluate the results from the OncoArray-TRICL data set. Another limitation came from over parameterizing our models, resulting in many multifactor cells with missing data [7]. This did not affect the classification of genotype combinations or identifying cross-validation consistency of the model, however, it could affect our estimation of the prediction error [7]. Future studies would need to address this limitation. Next, we applied our ES-MDR method to analyze survival outcomes using case–control studies, where estimating the age-specific incidence (e.g., age-of-onset) was not typically designed for case–control studies. On the other hand, cohort studies, which are designed for survival analyses, are expensive and require a great deal of follow-up time to obtain age-of-onset information. This could be one of the barriers in analyzing survival outcomes for large cohort studies; it could require a lot of time and resources to amass an extensive amount of data. In our study, we were able to analyze and identify potential genetic markers that predicted lung cancer risk using a large lung cancer GWAS consortium data, which could be followed up with further investigations for biological and functional significance. Due to fewer available observations of lung cancer age-of-onset among younger individuals, we were limited in our ability to predict lung cancer onset for individuals 40 years and younger. With continuous efforts in recruiting participants in the OncoArray-TRICL Consortium, we might find more cases among the early onset population to better predict lung cancer risk in the future. Finally, there were no available validation data to replicate our top SNP findings because these SNPs were not likely genotyped in other GWAS data sets. Currently, there are ongoing efforts to collect external data that will include genotyping of our top SNP findings for replication.

### Future studies

ES-MDR is a powerful alternative to Surv-MDR for identifying interactions, especially at the genome-wide scale. We demonstrated its ability to identify high-order genetic interactions in simulated and real data sets. Although ES-MDR addresses previous limitations of Surv-MDR and other MDR-like methods, there are ways in which this method can be improved. While ES-MDR had greatly improved computing efficiency, genome-wide scans for interactions will still require massive computing resources, especially to analyze higher-order interactions. It will be necessary to optimize the selection of SNPs in predictive models, for example, by selecting genes known to participate in biological and metabolic pathways [22]. This can improve the predictive ability of ES-MDR for two-, three, and multi-way interactions in a pathway analysis. Second, a future study may entail introducing variance back to Martingale Residuals by way of weighting each residual based on the time-to-event data. This can greatly improve our power for model selection without removing the efficiency of the algorithm.

## Conclusions

In summary, the ES-MDR method provides a way to analyze high-order interactions at the genome-wide scale to advance studies of genetic interactions. We developed a new method that efficiently captures non-linear and high-order interactions for time-to-event analysis. In general, ES-MDR has improved power performance relative to Surv-MDR using simulated data. Based on the noticeable trends, we are confident that with bigger sample sizes, ES-MDR will continue to significantly gain in power to detect functional interacting SNPs without inflating the type I error rate. Providing new and improved methods to analyze epistasis or gene interactions may offer new opportunities to not only explain the missing heritability for complex disease risk, but can also potentially detect new genetic determinants that is important for clinical utility such as disease diagnosis and prognosis.

## Data Availability

The datasets generated and/or analyzed during the current study are available in the NCBI dbGaP repository, dbGaP Study Accession: phs001273.v3.p2, website: https://www.ncbi.nlm.nih.gov/projects/gap/cgi-bin/study.cgi?study_id=phs001273.v3.p2.

## Abbreviations

GWAS: Genome-wide association studies
SNP: Single nucleotide polymorphism
ES-MDR: Efficient Survival Multifactor Dimensionality Reduction
HR: Hazard ratio
MDR: Multifactor Dimensionality Reduction
Surv-MDR: Survival MDR
QMDR: Quantitative MDR
TRICL: Transdisciplinary Research Into Cancer of the Lung
MAF: Minor allele frequency
Cox ph: Cox proportional hazards
GHz: Gigahertz
CPU: Central processing unit
GB: Gigabyte
TB: Terabyte
Lasso: Least absolute shrinkage and selection operator
ROC: Receiver operating characteristic
AUC: Area under the ROC curve
bp: Base pair
ncRNA: Noncoding transcript variant
UTR: Untranslated region
